# Susceptibility Profile and Epidemiological Cut-Off Values Are Influenced by Serotype in Fish Pathogenic *Streptococcus agalactiae*

**DOI:** 10.3390/antibiotics12121726

**Published:** 2023-12-13

**Authors:** Carlos Augusto Gomes Leal, Brendhal Almeida Silva, Salene Angelini Colombo

**Affiliations:** Department of Preventive Veterinary Medicine, Veterinary School, Federal University of Minas Gerais, Belo Horizonte 31270-901, Brazil; brendhal14@gmail.com (B.A.S.); angelinicolombo@gmail.com (S.A.C.)

**Keywords:** antibiotic, streptococcosis, fish, capsular serotyping, normalized resistance interpretation

## Abstract

*Streptococcus agalactiae* is a major health concern in tilapia farming worldwide. In contrast to the availability of susceptibility profile results, interpretative criteria for disk diffusion assays and the influence of serotypes on resistance profiles are not available. To address this, sixty isolates (thirty of each serotype, Ib and III) were evaluated using the disk diffusion assay against six antibiotics, and the epidemiological cut-off value (ECV) was calculated. All the isolates were classified as non-wild type (NWT) for sulfamethoxazole (SUT) and norfloxacin (NOR). The inhibition zones for oxytetracycline (OXY) and doxycycline (DOX) were largely distinct; all serotype Ib and III isolates were classified as wild-type (WT) and NWT, respectively. The results for serotype III of fish group B *Streptococcus* (GBS) were comparable to the NWT tetracycline profile of human GBS available in EUCAST, suggesting the presence of resistance mechanisms in these fish isolates. The calculation of the cut-off wild type (CO_WT_) values for OXY and DOX was appropriate for both serotypes. Differences between the distribution of florfenicol (FLO) and amoxicillin (AMO) were found, and we attribute this to the faster growth rate of serotype III, which promotes smaller inhibition zones. Therefore, using separate CO_WT_ for each serotype is necessary. In conclusion, the serotype of fish GBS affects its susceptibility profile, and it is recommended to use serotype-specific CO_WT_ values as interpretative criteria for disk diffusion assays against FLO and AMO.

## 1. Introduction

Nile tilapia (*Oreochromis niloticus*) is the second-most farmed fish worldwide [1], and has shown significant growth in recent decades owing to its high productivity and value as a source of protein and economic income [2]. However, recent outbreaks of *Streptococcus agalactiae* have caused major economic losses in the industry due to the resulting high morbidity and mortality rates in fish [3,4].

*S. agalactiae* is a Gram-positive bacterium, commonly known as group B *Streptococcus* (GBS), that affects a wide range of hosts, including mammals and aquatic animals. It is mainly associated with neonatal meningitis in humans, bovine mastitis, septicemia, and meningoencephalitis in fish [5,6]. This bacterium can be classified into 10 different serotypes (Ia, Ib, and II to IX) based on the specificity of the polysaccharide layer that surrounds it [7]. This layer determines the antigenic and structural uniqueness of the serotypes and can differentiate even in the epidemiology, virulence, and antimicrobial susceptibility profile [6,8,9,10,11,12,13,14].

Serotypes Ia, Ib, and III are the main causes of fish infections [13,15]. Serotype III has a history of causing outbreaks in Thailand and China [13,16], and was first reported on Brazilian tilapia farms in 2017 [9]. This serotype is associated with high zoonotic potential and has been linked to severe food-borne outbreaks associated with the consumption of raw fish in Singapore [17]. Unlike serotype Ib, which is a piscine-adapted lineage, serotype III seems to be a multi-host-adapted lineage capable of infecting both fish and mammals [18].

The routine use of antimicrobials on fish farms is a critical issue for food security and public health, as it can result in the emergence of resistant strains and control outbreaks caused by bacterial pathogens [19,20]. Despite being the main control measure for *S. agalactiae* in fish farms, the oral treatment with oxytretacycline and florfenicol has shown a significant increase in the resistance in recent years in the country [9,21,22,23].

Currently, qualitative and quantitative methods are available for determining antimicrobial susceptibility. Despite being a qualitative test, the diffusion disk assay remains one of the most versatile, low-cost, and reproducible tests that can be used routinely in laboratories [24,25]. The best predictors of antimicrobial outcomes are the cut-off points established by standards-setting organizations, such as the European Antimicrobial Susceptibility Testing Committee (EUCAST) and the Institute of Clinical and Laboratory Standards (CLSI). However, the data only pertains to bacteria found in mammals, not aquatic animals. In the absence of such determined cut-off points, normalized resistance interpretation (NRI) can be an efficient and sensitive method for distinguishing the deduced population from normal isolates from those with an increase in resistance and can, consequently, be useful in the surveillance of bacterial resistance in the host [24,26,27].

Previous studies have evaluated the antimicrobial susceptibility of GBS in fish isolates and demonstrated resistance to different classes of antimicrobials [9,10,13,23]. Some studies have suggested that the susceptibility profile is related to the GBS serotype involved in infection. Serotype III demonstrated a greater proportion of antibiotic resistance than other serotypes in infections in fish [9,10]. A major problem is that the criteria for interpreting the epidemiological cut-off point available in the literature are variable, erroneous, and often extrapolated from human isolate data to aquatic animal bacteria. It is known that, although it is the same species, the GBS serotypes associated with infection in fish can present different phenotypic traits as well as antibiotic resistance profiles, which can influence the results of antimicrobial sensitivity tests when approached as a single type. In this context, there are still no data on the resistance profile of different serotypes that cause streptococcosis in fish. Thus, the aim of this study was to evaluate the susceptibility profile of different serotypes of *S. agalactiae* in fish and to calculate epidemiological cut-off points (ECV).

## 2. Results

### 2.1. Sulfamethoxazole 25 and Norfloxacin 10 Disk Studies

All isolates of both serotypes were categorized as NWT for sulfamethoxazole and norfloxacin.

### 2.2. Oxytetracycline 30 and Doxycycline 30 Disk Studies

**Figure 1:**
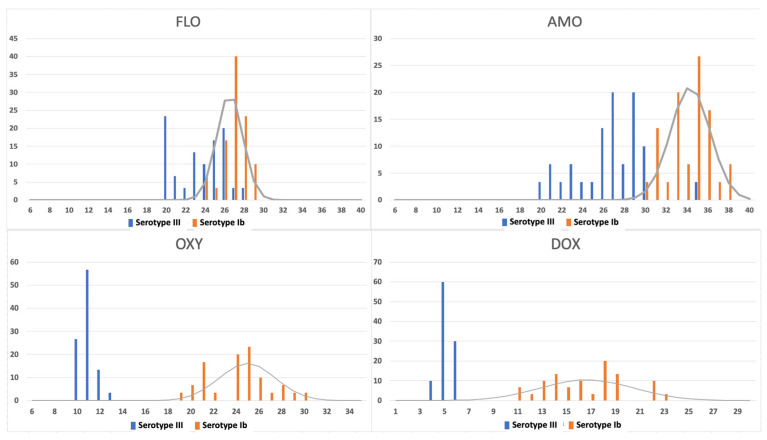
Histograms of the inhibition zone for *S. agalactiae* serotypes III (blue bars) and Ib (orange bars) against oxytetracycline (OXY), doxycycline (DOX), florfenicol (FLO), and amoxicillin AMO (X axis: inhibition zones in mm; Y axis: number of isolates). Superimposed on the histograms are the curves calculated for the distributions according to the standardized interpretation method.

Figure 1 shows the distribution of inhibition zones obtained from the evaluated isolates. Different frequency distributions were observed in the inhibition zones for OXY and DOX. These different patterns in the histograms were attributed to the low-frequency distribution of serotype III isolates in relation to serotype Ib. It is observed that, when analyzed separately, the CO_WT_ values calculated from data from serotypes Ib and III for OXY were ≤19 mm and ≤10 mm and for DOX were ≤12.0 mm and ≤8.0 mm, respectively. When analyzed together, the cut-off value of the two serotypes is ≤19.0 mm for OXY and ≤12 mm for DOX (Table 1).

The combined analysis of the isolates showed stronger data, so all isolates were classified as wild-type for serotype Ib and non-wild-type for serotype III, based on the CO_WT_ for DOX and OXY (Table 2).

To better investigate the use of epidemiological cut-off values for combined serotypes, the data of this study were compared with the susceptibility data of minocycline and tetracycline for *S. agalactiae* and *S. dysgalactiae* released by EUCAST, as described in Table 3. The results for serotype III were similar to the NWT tetracycline profile of human GBS available in the EUCAST, confirming that the combined isolation approach is appropriate for the epidemiological cut-off point.

### 2.3. Florfenicol 30 and Amoxicillin 10 Disk Studies

**Figure 2:**
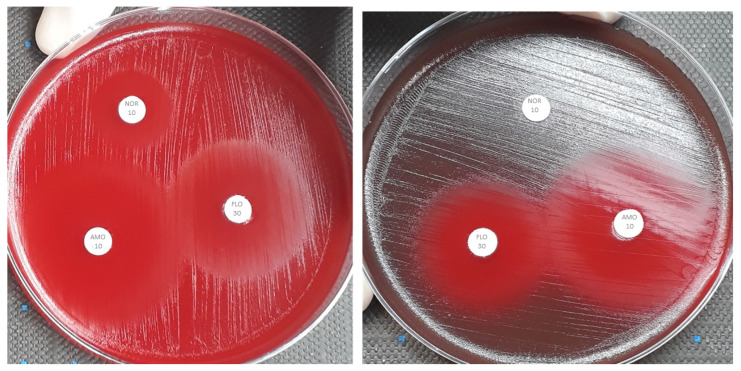
Disk diffusion test plates with GBS serotype Ib (**left**) and III (**right**) showing bacterial growth and inhibition zones for amoxicillin (AMO 10), florfenicol (FLO 30), and norfloxacin (NOR 10). The greater and faster growth of the serotype III isolate suggests that the phenotypic characteristics of these strains may influence the test results.

The frequency distributions of serotypes Ib and III for FLO and AMO differed. When analyzed together, the cut-off values of the two serotypes were ≤23.0 mm for FLO and ≤30 mm for AMO. When analyzed separately, the CO_wt_ for serotypes Ib and III for FLO were ≤23 mm and ≤21 mm and for AMO were ≤30.0 mm and ≤23.0 mm, respectively (Table 1). Figure 2 shows that serotype III grows faster and creates a smaller inhibition zone than serotype Ib. Although this does not indicate antimicrobial resistance, combining it with serotype Ib can lead to that result.

In summary, five isolates were considered non-wild-type for FLO (*n* = 3) and AMO (*n* = 4). In addition, all serotype III isolates were classified as multi-resistant bacteria. All zone diameters obtained for *Escherichia coli* ATCC 25922 were found at acceptable quality control intervals, as determined by CLSI; therefore, our results were valid.

## 3. Discussion

In Brazil, Nile tilapia (*Oreochromis niloticus*) is the main aquatic host for *S. agalactiae* and outbreaks of the disease have been described in several Brazilian states, and are one of the most important factors limiting the expansion of tilapia farming in Brazil [9,15,18,28,29].

In cases of outbreaks caused by *S. agalactiae*, mortality can reach up to 90% of the herd [30,31]. The most efficient measure to control this disease is the use of antimicrobials [10]. The efficacy of some antibiotics in reducing mortality during tilapia outbreaks has been found to be inadequate, leading to recurrent infections even after treatment [9,22].

Resistance to sulphonamide and fluoroquinolones has already been described in fish GBS strains [9,10,23,32] and humans [32,33,34]. In contrast, a study carried out in Thailand obtained results similar to those of sulfamethoxazole, but most isolates showed sensitivity to norfloxacin [35].

In Brazil, only oxytetracycline and florfenicol are authorized for use in aquaculture and are widely used in the treatment of streptococcosis in Nile tilapia. The widespread use of OXY as a prophylactic treatment may also have contributed to an increase in bacterial resistance in fish [36]. Some strains of GBS have been described as resistant to OXY in Nile tilapia and Amazon catfish (*Leiaurius marmoratus × Pseudoplatystoma corruscans*) in the country [9,23]. Although such antimicrobials are effective in reducing herd mortality, these animals can still eliminate the pathogen in the environment [31]. According to our findings, emerging GBS strains of serotype III were found to be resistant to several antibiotics, including ampicillin, norfloxacin, aminoglycosides, fluoroquinolone, sulfamethoxazole, and tetracycline [9]. In the present study, the NWT phenotype may be due to the resistance mechanisms of these antimicrobials. Bergal et al. [37] indicated that the tetM and tetO genes may be involved in this process. Tetracycline resistance is governed by tet genes, which are compromised in the drug’s active efflux, ribosomal protection, or enzymatic modification [38].

A recent study observed the presence of this gene in GBS isolates from fish and humans in the same region in China, correlating the observed phenotypic resistance with the presence of specific genes of tet [32]. In addition, reduced susceptibility to human GBS has also been described in Brazil for tetracyclines [39,40,41,42], as well as the resistance gene tet being detected in strain resistance in humans and cows [40]. EUCAST disk data for *S. agalactiae* were available for tetracycline and minocycline and MIC data for tetracycline and doxycycline. It is interesting to note that the application of ECOFF values accepted by EUCAST to the NWT frequency was approximately 80% in all four EUCAST groups.

According to EUCAST, the TET and MIN zones classified as NWT for *S. agalactiae* are similar to the OXY and DOX zones in the present study. Thus, we argue that all serotype III isolates analyzed in this study may also be NWT. It should be noted that EUCAST data were obtained using different agents, and the tests were conducted at 35 °C. Table 3 demonstrates significant similarities between the NRI analysis parameters of EUCAST data distribution and the combined serotypes III and Ib obtained in the study, despite some differences. This analysis suggests that the use of epidemiological cut-off values (CO_WT_) for DOX and OXY calculated from all isolates of *S. agalactiae*, regardless of serotype, would generate a more appropriate classification, similar to that of the isolates generated by the application of ECOFF values from EUCAST. Thus, the CO_WT_ for OXY and DOX calculated for both serotypes was adequate in this study.

Differences between the distributions of FLO and AMO were observed. This was probably associated with distinct phenotypic traits of the serotypes, since a faster growth rate is presented by serotype III, which promotes smaller inhibition zones. Therefore, a separate CO_WT_ for each serotype is meaningful. In routine laboratory observations, serotype III is phenotypically very different from serotype Ib, especially in terms of growth. Serotype Ib shows slower growth in culture, as compared to other GBS serotypes, which initially promoted the classification of that serotype as another species of the genus *Streptococcus*, as *S. difficile*. However, despite the noticeable phenotypic differences, further studies have confirmed that these strains belong to the species *S. agalactiae* and can be attributed to serotype Ib [42,43]. It is reasonable to assume that the difference in the antibiogram for disc diffusion between serotypes Ib and III could be explained by the difference in their growth rates. Therefore, the fastest-growing serotype III promoted the smallest zone. The factors affecting zones in disk diffusion assays are complex and have received limited attention in recent decades. It is reasonable to suggest that WT strains which have slower growth rates would tend to have larger zones than WT strains which have the same susceptibility but faster growth rates. In writing on the theory of disc diffusion zones, Cooper [44] identified the bacterial growth rate as one of the factors influencing disk diffusion assay results. Similar observations were noted by Smith et al. [45]. The author analyzed some distributions of strains of *Aeromonas salmonicida* and suggested that the slow-growing strains had larger zones than those that showed common growth rates. Based on the present results, the calculation of CO_WT_ for AMO and FLO for each serotype should be more appropriate for tilapia pathogenic *S. agalactiae*.

Florfenicol is recommended for use in aquaculture because of its effectiveness and relative safety [46]. In the present study, 10% of the GBS isolates from serotype III (3/30) were classified as NWT for this drug. This is noteworthy, as previous studies on this serotype demonstrate, that there is no resistance to this class of antibiotics in Brazilian tilapia bacteria. Previous studies evaluating piscine GBS serotype III have demonstrated the sensitivity of these isolates to FLO [10,33]. Conversely, serotype II GBS isolated from Amazon catfish in Brazil demonstrated NWT against FLO [23]. In addition, four samples were considered NWT for amoxicillin. Though few studies have described resistance to amoxicillin in Nile tilapia infected with *S. agalactiae*, previous studies have found similar results in Amazon catfish [23]. The results obtained in this study and those of Tavares et al. [23] show the circulation of FLO- and AMO-resistant GBS strains in Brazilian fish farming, which is an important health issue for the sector.

The emergence of multidrug-resistant (MDR) strains is a major global problem in veterinary medicine and human health [47]. An isolate can be defined as multidrug-resistant (MDR) if it is resistant to three or more classes of antimicrobials. In the present study, some GBS serotype III isolates were classified as MDR, as they were classified as NWT to three antimicrobial classes. Similar results have previously been described by Chideroli et al. [9]. A recent study has described multidrug-resistant GBS serotype III isolates from fish and humans in China [10,32]. Antimicrobial resistance in fish pathogenic GBS serotype III seems to be an important health issue for fish farming worldwide.

A recent study demonstrated a high prevalence of virulence genes associated with serotype III, as well as high virulence when compared to strains of serotype Ia of fish in Thailand [48]. It is necessary to investigate the genetic characteristics of Brazilian GBS isolates of serotypes Ib and III related with virulence and antibiotic resistance in fish.

## 4. Materials and Methods

### 4.1. Bacterial Strains

Sixty strains of *S. agalactiae* isolated from diseased Nile tilapia that belonged to the serotypes Ib (*n* = 30) and III (*n* = 30) were evaluated. The isolates were obtained from the culture collection of the Laboratory of Routine Bacteriology, Veterinary School of the Federal University of Minas Gerais (UFMG).

These strains originated from outbreaks at Nile tilapia production farms in eight different states in Brazil (Table 4). The animals were sent to the Laboratory of Routine Bacteriology for bacteriological diagnosis. Smears taken from brain and kidney samples were aseptically cultured on 5% horse blood agar (HBA) and incubated at 28 °C for 72 h. Suspected isolates of *S. agalactiae* have previously been identified using phenotypic and molecular methods [15]. The isolates were then stored in brain and heart infusion broth (BHI) with 15% of glycerol at −80 °C until use.

### 4.2. Antimicrobial Susceptibility Testing

The disk diffusion test of the isolates and reference strains was performed according to the VET03-A guidelines established by the Clinical and Laboratory Standard Institute^®^ [49], with adaptations recommended for streptococcal bacteria (Group 4). Six antimicrobials were used: amoxicillin (AMO, 10 μg); doxycycline (DOX, 30 μg); florfenicol (FLO, 30 μg); norfloxacin (NOR, 10 μg); oxytetracycline (OXY, 30 μg); and sulfamethoxazole with trimethoprim (SUT, 25 μg). The discs were acquired from Oxoid ™ (Thermo Scientifics, Wilmington, NC, USA). The *Streptococcus agalactiae* isolates was thawed, cultured on 5% horse blood agar, and incubated at 28 °C for 24 h. After incubation, each strain was harvested and suspended in sterile saline to achieve an absorbance value of 625 nm between 0.08 and 0.13 (DO625). Muller–Hinton agar plates enriched with 5% defibrinated sheep blood were inoculated with bacteria by scraping with sterile swabs. Then, the plates were incubated at 28 °C for 24 h. In addition, the strain of *Escherichia coli* ATCC 25922 was used as a quality control strain. The inhibition zones for both serotypes were measured 24 and 48 h after incubation. One person evaluated all antibiogram plates to reduce observer bias in the size of the zones of inhibition.

### 4.3. Statistical Analysis

Normalized resistance interpretation (NRI) was conducted using the method developed by Kronvall [24] and Kronvall et al. [27], according to the adaptation by Smith et al. [47]. This method calculates epidemiological cut-off values (CO_WT_) to classify a species’ isolates as fully susceptible wild type (WT) or non-wild-type (NWT) based on their susceptibility. NWT isolates show significantly reduced susceptibility compared to WT within the population. NRI analyses of the inhibition zone data were performed using automated Excel spreadsheets available at http://www.bioscand.se/nri/ (accessed on 28 February 2022). The epidemiological cut-off values for defining susceptible strains were set at 2.5 standard deviations below the normalized average [45]. In this study, strains that generated zones equal to or greater than the NRI limit for susceptible strains were classified as wild-type (WT), and those that generated smaller zones were classified as non-wild-type (NWT). In addition, the epidemiological cut-off values for serotypes of tetracyclines (OXY and DOX) were compared with susceptibility data for *S. agalactiae* and *S. dysgalactiae* released by EUCAST for humans [50].

## 5. Conclusions

In conclusion, the serotype of fish GBS influences the susceptibility profile, and distinct CO_WT_ according to the serotype should be used as interpretative criteria for disk diffusion assays against FLO and AMO.

## Figures and Tables

**Table 1:** Epidemiological cut-off (CO_WT_) values (mm) calculated by NRI for *Streptococcus agalactiae* serotype Ib and III separated (WT mean) with standard deviations and combined (Comb) for DOX, OXY, FLO, and AMO antibiotics.

	Wild-type (WT) Mean			Wild-type (WT) SD			CO_WT_		
	III	Ib	Comb	III	Ib	Comb	III	Ib	Comb
DOX	10	22	22	0.74	3.79	3.79	8	12	12
OXY	10	25	25	0.56	2.47	2.47	10	19	19
FLO	25	27	27	1.4	1.11	1.3	21	24	23
AMO	28	34	35	1.96	1.91	1.88	23	30	30

AMO = amoxicillin, 10 µg; DOX = doxycycline, 30 µg; FLO = florfenicol, 30 µg; OXY = oxytetracycline, 30 µg.

**Table 2:** Antibiotic susceptibility patterns of *Streptococcus agalactiae* of serotype Ib and III strains from diseased Nile tilapia. The inhibition zone diameters (in mm) of each strain were measured following growth in Muller–Hinton agar enriched with 5% of defibrinated sheep blood at 28 °C for 24 h. WT (wild-type) denoted susceptible strains; NWT (non-wild-type) represented resistant strains.

Strain	Serotype III				Strain	Serotype Ib			
	OXY	DOX	AMO	FLO		OXY	DOX	AMO	FLO
492/19	11 (NWT)	11 (NWT)	27 (WT)	25 (WT)	189/17	26 (WT)	27 (WT)	33 (WT)	27 (WT)
118/17	11 (NWT)	10 (NWT)	29 (WT)	20 (NWT)	58/17	30 (WT)	26 (WT)	30 (WT)	26 (WT)
149/17	11 (NWT)	10 (NWT)	39 (WT)	26 (WT)	86/17	22 (WT)	18 (WT)	34 (WT)	27 (WT)
294/18	10 (NWT)	11 (NWT)	22 (NWT)	21 (WT)	68/17	24 (WT)	23 (WT)	30 (WT)	26 (WT)
237/18	11 (NWT)	9 (NWT)	24 (WT)	21 (WT)	311/18	25 (WT)	23 (WT)	33 (WT)	29 (WT)
325/18	11 (NWT)	11 (NWT)	22 (NWT)	21 (WT)	366/18	25 (WT)	23 (WT)	34 (WT)	28 (WT)
328/18	11 (NWT)	10 (NWT)	25 (WT)	21 (WT)	310/18	26 (WT)	22 (WT)	32 (WT)	28 (WT)
27/17	11 (NWT)	10 (NWT)	26 (WT)	21 (WT)	365/18	25 (WT)	19 (WT)	35 (WT)	28 (WT)
215/18	11 (NWT)	9 (NWT)	22 (NWT)	20 (NWT)	251/18	22 (WT)	24 (WT)	31 (WT)	25 (WT)
117/17	11 (NWT)	10 (NWT)	22 (NWT)	20 (NWT)	393/18	23 (WT)	22 (WT)	35 (WT)	26 (WT)
327/18	10 (NWT)	10 (NWT)	29 (WT)	24 (WT)	339/18	23 (WT)	21 (WT)	29 (WT)	25 (WT)
239/18	10 (NWT)	10 (NWT)	30 (WT)	27 (WT)	421/18	28 (WT)	26 (WT)	32 (WT)	26 (WT)
306/18	10 (NWT)	10 (NWT)	31 (WT)	26 (WT)	496/19	25 (WT)	22 (WT)	31 (WT)	24 (WT)
399/18	10 (NWT)	11 (NWT)	33 (WT)	24 (WT)	472/19	27 (WT)	28 (WT)	40 (WT)	27 (WT)
330/18	11 (NWT)	10 (NWT)	32 (WT)	28 (WT)	495/19	28 (WT)	25 (WT)	34 (WT)	28 (WT)
400/18	11 (NWT)	10 (NWT)	31 (WT)	27 (WT)	482/19	27 (WT)	24 (WT)	32 (WT)	29 (WT)
487/19	11 (NWT)	10 (NWT)	34 (WT)	27 (WT)	514/19	28 (WT)	26 (WT)	35 (WT)	28 (WT)
488/19	12 (NWT)	11 (NWT)	34 (WT)	29 (WT)	502/19	26 (WT)	28 (WT)	40 (WT)	29 (WT)
03/17	10 (NWT)	10 (NWT)	32 (WT)	27 (WT)	516/19	26 (WT)	23 (WT)	33 (WT)	28 (WT)
26/17	10 (NWT)	10 (NWT)	34 (WT)	28 (WT)	505/19	25 (WT)	20 (WT)	37 (WT)	28 (WT)
212/18	11 (NWT)	10 (NWT)	26 (WT)	23 (WT)	506/19	21 (WT)	18 (WT)	32 (WT)	28 (WT)
334/18	10 (NWT)	10 (NWT)	27 (WT)	22 (WT)	265/18	24 (WT)	23 (WT)	35 (WT)	29 (WT)
240/18	11 (NWT)	10 (NWT)	28 (WT)	24 (WT)	523/19	22 (WT)	19 (WT)	33 (WT)	26 (WT)
491/19	11 (NWT)	11 (NWT)	29 (WT)	25 (WT)	513/19	25 (WT)	24 (WT)	28 (WT)	24 (WT)
219/18	12 (NWT)	10 (NWT)	29 (WT)	27 (WT)	390/18	29 (WT)	23 (WT)	33 (WT)	30 (WT)
322/18	10 (NWT)	11 (NWT)	26 (WT)	26 (WT)	88/17	21 (WT)	18 (WT)	35 (WT)	26 (WT)
137/17	12 (NWT)	11 (NWT)	30 (WT)	26 (WT)	246/18	23 (WT)	18 (WT)	31 (WT)	29 (WT)
213/19	12 (NWT)	10 (NWT)	27 (WT)	23 (WT)	253/18	25 (WT)	23 (WT)	37 (WT)	27 (WT)
397/18	12 (NWT)	9 (NWT)	25 (WT)	24 (WT)	164/17	22 (WT)	17 (WT)	34 (WT)	27 (WT)
223/18	13 (NWT)	10 (NWT)	34 (WT)	27 (WT)	160/17	22 (WT)	19 (WT)	34 (WT)	29 (WT)

AMO = amoxicillin, 10 µg; DOX = doxycycline, 30 µg; FLO = florfenicol, 30 µg; OXY = oxytetracycline, 30 µg.

**Table 3:** Epidemiological cut-off (CO_WT_) values (mm) calculated by NRI for GBS serotype of fish [Ib + III] compared to the human susceptibility data for *S. agalactiae* and *S. dysgalactiae*, available in EUCAST for tetracyclines.

Species	Agent	Source	Wild-type Mean	Wild-type SD	CO_WT_
*S. agalactiae*	DOX	III + Ib (Present study)	22	3.79	12
	OXY		25	2.45	19
	TET	EUCAST **	22	2.19	20
	MIN		25	2.47	19
*S. dysgalactiae*	TET		27	2.03	21

DOX = doxycycline, 30 µg; OXY = oxytetracycline, 30 µg; TET = tetracycline, 30 µg; MIN = minocycline, 10 μg; ** European Committee on Antimicrobial Susceptibility Testing.

**Table 4:** Strains, year, serotype, and origin of *Streptococcus agalactiae* isolates used in this study.

Strain	Year	Serotype	State	City
SA 189/17	2017	Ib	Pernambuco	Jatobá
SA 58/17	2017	Ib	Pernambuco	Jatobá
SA 86/17	2017	Ib	Ceará	Fortaleza
SA 68/17	2017	Ib	Pernambuco	Jatobá
SA 88/17	2017	Ib	Ceará	Fortaleza
SA 164/17	2017	Ib	São Paulo	Rifaina
SA 160/17	2017	Ib	São Paulo	Rifaina
SA 311/18	2018	Ib	São Paulo	Santa Fé do Sul
SA 366/18	2018	Ib	São Paulo	Santa Fé do Sul
SA 310/18	2018	Ib	São Paulo	Santa Fé do Sul
SA 365/18	2018	Ib	São Paulo	Santa Fé do Sul
SA 251/18	2018	Ib	São Paulo	Rio Grandinho
SA 393/18	2018	Ib	Minas Gerais	Carmo do Rio Claro
SA 339/18	2018	Ib	Bahia	Paulo Afonso
SA 421/18	2018	Ib	Minas Gerais	Uberlandia
SA 265/18	2018	Ib	Bahia	Paulo Afonso
SA 390/18	2018	Ib	Minas Gerais	Carmo do Rio Claro
SA 246/18	2018	Ib	São Paulo	Santa Fé do Sul
SA 253/18	2018	Ib	São Paulo	Santa Fé do Sul
SA 496/19	2019	Ib	Bahia	Paulo Afonso
SA 472/19	2019	Ib	São Paulo	Jau
SA 495/19	2019	Ib	Bahia	Paulo Afonso
SA 482/19	2019	Ib	Minas Gerais	Alfenas
SA 514/19	2019	Ib	Mato Grosso	Chapada dos Guimaraes
SA 502/19	2019	Ib	Mato Grosso do Sul	Selvíria
SA 516/19	2019	Ib	Paraná	Toledo
SA 505/19	2019	Ib	São Paulo	Santa Fé do Sul
SA 506/19	2019	Ib	São Paulo	Santa Fé do Sul
SA 523/19	2019	Ib	Minas Gerais	Carmo do Rio Claro
SA 513/19	2019	Ib	Mato Grosso	Chapada dos Guimaraes
SA 118/17	2017	III	Bahia	Valença
SA 149/17	2017	III	Bahia	Valença
SA 27/17	2017	III	Bahia	Gloria
SA 117/17	2017	III	Bahia	Valença
SA 03/17	2017	III	Pernambuco	Itacarube
SA 26/17	2017	III	Bahia	Gloria
SA 137/17	2017	III	Bahia	Valença
SA 294/18	2018	III	Pernambuco	Jatobá
SA 237/18	2018	III	Pernambuco	Santo Antonio
SA 325/18	2018	III	Piaui	Guadalupe
SA 328/18	2018	III	Piaui	Guadalupe
SA 215/18	2018	III	Bahia	Valença
SA 327/18	2018	III	Piaui	Guadalupe
SA 239/18	2018	III	Pernambuco	Santo Antonio
SA 306/18	2018	III	Bahia	Gloria
SA 399/18	2018	III	Maranhao	São Joao do Maranhão
SA 330/18	2018	III	Bahia	Paulo Afonso
SA 400/18	2018	III	Maranhao	São Joao do Maranhão
SA 212/18	2018	III	Bahia	Valença
SA 334/18	2018	III	Bahia	Paulo Afonso
SA 240/18	2018	III	Pernambuco	Santo Antonio
SA 219/18	2018	III	Alagoas	Coruripe
SA 322/18	2018	III	Piaui	Guadalupe
SA 397/18	2018	III	Ceará	Fortaleza
SA 223/18	2018	III	Alagoas	Coruripe
SA 492/19	2019	III	Bahia	Paulo Afonso
SA 487/19	2019	III	Alagoas	Piranhas
SA 488/19	2019	III	Alagoas	Piranhas
SA 491/19	2019	III	Bahia	Paulo Afonso
SA 213/19	2019	III	Bahia	Valença

## Data Availability

Data are contained within the article.
